# Student well-being during dedicated preparation for USMLE Step 1 and COMLEX Level 1 exams

**DOI:** 10.1186/s12909-021-03055-2

**Published:** 2022-01-04

**Authors:** Sean Tackett, Maniraj Jeyaraju, Jesse Moore, Alice Hudder, Sandra Yingling, Yoon Soo Park, Mark Grichanik

**Affiliations:** 1 Johns Hopkins Bayview Medical Center, 5200 Eastern Ave, MFL Center Tower Suite 2300, Baltimore, MD 21224 USA; 2 University of Maryland School of Medicine, Baltimore, USA; 3 Larner College of Medicine at the University of Vermont, Burlington, USA; 4 Lake Erie, College of Osteopathic Medicine, Erie, USA; 5 University of Illinois College of Medicine, Chicago, USA; 6 Harvard Medical School and Massachusetts General Hospital, Boston, USA; 7 David Geffen School of Medicine at UCLA, Los Angeles, USA

**Keywords:** USMLE step 1, COMLEX level 1, Well-being, Medical students

## Abstract

**Background:**

Nearly all U.S. medical students engage in a 4–8 week period of intense preparation for their first-level licensure exams, termed a “dedicated preparation period” (DPP). It is widely assumed that student well-being is harmed during DPPs, but evidence is limited. This study characterized students’ physical, intellectual, emotional, and social well-being during DPPs.

**Methods:**

This was a cross-sectional survey sent electronically to all second-year students at four U.S. medical schools after each school’s respective DPP for USMLE Step 1 or COMLEX Level 1 in 2019. Survey items assessed DPP characteristics, cost of resources, and perceived financial strain as predictors for 18 outcomes measured by items with Likert-type response options. Open-ended responses on DPPs’ influence underwent thematic analysis.

**Results:**

A total of 314/750 (42%) students completed surveys. DPPs lasted a median of 7 weeks (IQR 6–8 weeks), and students spent 70 h/week (IQR 56–80 h/week) studying. A total of 62 (20%) reported experiencing a significant life event that impacted their ability to study during their DPPs. Most reported 2 outcomes improved: medical knowledge base (95%) and confidence in ability to care for patients (56%). Most reported 9 outcomes worsened, including overall quality of life (72%), feeling burned out (77%), and personal anxiety (81%). A total of 25% reported paying for preparation materials strained their finances. Greater perceived financial strain was associated with worsening 11 outcomes, with reported amount spent associated with worsening 2 outcomes. Themes from student descriptions of how DPPs for first-level exams influenced them included (1) opportunity for synthesis of medical knowledge, (2) exercise of endurance and self-discipline required for professional practice, (3) dissonance among exam preparation resource content, formal curriculum, and professional values, (4) isolation, deprivation, and anguish from competing for the highest possible score, and (5) effects on well-being after DPPs.

**Conclusions:**

DPPs are currently experienced by many students as a period of personal and social deprivation, which may be worsened by perceived financial stress more than the amount of money they spend on preparation materials. DPPs should be considered as a target for reform as medical educators attempt to prevent student suffering and enhance their well-being.

**Supplementary Information:**

The online version contains supplementary material available at 10.1186/s12909-021-03055-2.

## Background

The potential for the first-level medical licensure exams in the U.S. to cause stress for students was apparent in the early 1980s, after more medical schools began requiring students to pass the National Board of Medical Examiners (NBME) comprehensive Part I exam (the predecessor to United States Medical Licensing Exam (USMLE) Step 1) before moving on to their clerkships [1]. NBME Part I and II exams were not high on the list of factors used in residency selection then [2], but by 2008, when the first survey of residency program directors was conducted by the National Resident Matching Program (NRMP), USMLE Step 1 and the equivalent exam for osteopathic schools, Comprehensive Osteopathic Medical Licensing Examination (COMLEX) Level 1, were collectively the top-ranked factor used by programs when selecting which applicants to interview [3]. USMLE Step 1 and COMLEX Level 1 have remained at the top of the list in NRMP program director surveys since then. The pressure that students had felt to compete and perform well on Step 1, and the toll it was taking on them personally, were recently described in articles on “Step 1 climate” [4] and “Step 1 mania” [5]. The sponsors of the USMLE became sufficiently concerned about the potential impact of Step 1 on student well-being to organize the Invitational Conference on USMLE Scoring (INCUS) in March 2019, which resulted in the announcement that USMLE Step 1 will change to pass/fail reporting after January 1, 2022 [6]. The National Board of Osteopathic Medical Examiners announced in December 2020 that it would also change COMLEX Level 1 to pass/fail reporting beginning in May 2022 [7].

Many hope that the change to pass/fail reporting for U.S. first-level licensure exams will relieve stress on medical students. However, evidence suggests that the stress may instead be deferred to second-level licensure exam performance. In a national survey of U.S. program directors conducted after the announcement that Step 1 would become pass/fail, 77% of respondents indicated that they would require USMLE Step 2 Clinical Knowledge (CK) three-digit scores to be reported, and 81% would increase the emphasis on USMLE Step 2 CK scores when selecting applicants [8].

It may also be a testament to the pervasiveness of Step 1-related stress that, despite a significant body of literature related to USMLE exams [9], few investigators have formally studied Step 1’s influence on student well-being. Amid an array of commentaries [4, 5, 10, 11], we found only a statement describing an unpublished survey at one medical school that “concern that curricular content did not match what [students] were expected to know to perform well on the USMLE test … caused anxiety in more than 70% of students.” [10] A recent study at one U.S. medical school indicated that large proportions of its students attributed negative well-being to USMLE Step 1 preparation [12].

Nearly all medical students engage in a period of intense preparation for their first-level licensure exams, which we will refer to as a “dedicated preparation period” (DPP). This is typically a four- to eight-week period before clerkships in which students study for their exams, free from scheduled curricular activities. Schools that allow students to take these exams during clerkship years also typically provide dedicated time to prepare [13]. Studies related to dedicated preparation for USMLE or COMLEX exams have reported on DPP duration, study activities, and study resources [12, 14–31]. DPPs are a time in which many students attempt to have a sole focus on their upcoming licensure exam and a time in which exam-related stress is likely to be most intense; however, no study to our knowledge has focused on student well-being during DPPs.

Our primary goal in this study was to characterize student experiences during DPPs for USMLE Step 1 and COMLEX Level 1 at four U.S. medical schools, with a focus on the physical, intellectual, emotional, and social aspects of students’ well-being [32]. A secondary goal was to examine relationships between the financial factors related to exam preparation and student well-being, given the increasing attention being paid to the cost of preparing for and taking USMLE and COMLEX exams in the setting of increasing student debt.

## Methods

### Subjects and setting

This study was done on data collected through a larger cross-sectional survey (complete instrument included as supplementary material). It was sent electronically to all students in their second year of study at four U.S. medical schools, three allopathic schools (Larner College of Medicine at the University of Vermont, Rush Medical College, University of Illinois College of Medicine) and the largest campus at one osteopathic school (Lake Erie College of Osteopathic Medicine). The survey was sent in 2019 after each school’s respective DPP for USMLE Step 1 or COMLEX Level 1. All four schools required their respective first-level exams to be taken before students began their clerkships. Taking USMLE Step 1 was optional for students at the osteopathic school, although a majority (55%) among those sent the survey chose to take it; students at that school usually would take USMLE Step 1 at the end of their DPP and around the same time as they took COMLEX Level 1. All schools provided students practice tests developed by NBME and additional commercial resources to aid in preparation. The time given to students for DPPs ranged from six to eight weeks.

Appropriate ethical approval was obtained at each institution.

### Survey composition

Survey content was based on a review of the literature and our team’s experiences in counseling students on exam preparation at our respective institutions. We included items assessing time spent in DPPs, study resources used, confidence in resources, cost of resources, and perceived financial strain. These items were conceptualized as predictors of 18 items that measured outcomes of DPPs.

Sixteen of the items measuring outcomes asked about changes due to DPPs. Respondents were asked along a five-point Likert-type scale (1 = got much worse, 5 = got much better) to what extent exam preparation influenced: four items about professional development (medical knowledge base, confidence in ability to care for patients, confidence medical school was the right choice, confidence to be competitive for their specialty choice), two items about relationships (with colleagues, with loved ones), two items about quality of life (balance in personal and professional life, overall quality of life), one item about personal anxiety levels, and one item about feeling burned out from medical school. Six items asked along a five-point Likert-type scale (1 = much less, 5 = much more), compared to usual, how much respondents engaged in healthy behaviors (sleeping, eating healthy foods, exercising, spending time with friends, spending time with family, taking time away from academic responsibilities). All 16 of these items included a neutral option (e.g., “no change,” “about the same as usual”).

Two of the 18 outcome items asked how often during the DPP respondents either felt burned out from work and studying or bothered by feeling down, depressed, or hopeless (1 = never, 6 = every day).

Items related to confidence were adapted from a study on medical student doubt [33]. Items related to healthy behaviors were adapted from a study of internal medicine residents [34]. Items on quality of life, burnout, and feeling depressed were based on studies of medical learners using similar items [35–38]. All items were piloted with medical students who had previously completed their DPPs and revised for clarity and relevance prior to survey administration to our full sample.

Students have major life events while in medical school [39], but the impact of these events is understudied. Surveys included an item asking if respondents experienced “a significant life event that you believe impacted your ability to study.” If students answered yes, they were asked to categorize the event as (1) something that happened to a family or friend, (2) personal illness, (3) financial matter, and/or (4) an academic/extracurricular commitment. Respondents were also asked to describe the event.

Three additional open-ended items asked students (1) “Would you please share how preparing for USMLE Step 1/COMLEX Level 1 influenced you personally and professionally?”; (2) “How did you feel after you completed your exam? What was it like to have the exam behind you?”; and (3) “What did you do after you took your exam?”

### Data analysis

We first calculated descriptive statistics. In bivariate analyses, we created quartiles for continuous predictor variables because they were not normally distributed. We dichotomized all outcomes variables based on how we hypothesized they would change as a result of the DPP. We aggregated “got much better” and “somewhat better” (vs the 3 other response options) for the four professional development items. We dichotomized the other 12 items measuring changes to well-being by aggregating “got much worse” and “somewhat worse” (vs the 3 other response options). We dichotomized healthy behavior items by aggregating “much less” and “somewhat less” (vs the 3 other response options) and refer to these as “worse.” Burnout and depressive symptoms were determined to be present if they occurred weekly or more often during the DPP. We used chi-squared tests to examine differences in rates of outcomes across schools. For analyses of predictors for each outcome, we performed logistic regressions adjusting for clustering within schools and report unadjusted *p*-values. We set statistical significance at alpha < .05. When interpreting results, we applied Bonferroni correction for 18 comparisons, which requires an unadjusted *p* value of < .0027 for an alpha of < .05. Stata 13 (StataCorp. 2013. Stata Statistical Software: Release 13. College Station, TX, USA. StataCorp LP) was used for data analysis.

Responses to open-ended prompts on surveys often do not lend themselves to formal qualitative analysis [40]. However, students completing this survey often provided detailed responses to the open-ended item about the personal and professional impact of preparation for exams. Two authors coded responses for themes independently using an editing analysis method. Responses to other open-ended items were less descriptively rich but were likewise analyzed independently by two co-authors to summarize their contents.

## Results

A total of 326/750 (43%) students responded to surveys, with response rates ranging from 37 to 53% across schools. Complete data were available for 314/750 (42%). Before DPPs, students spent a median of 10 h/week (interquartile range (IQR) 5–21 h/week) preparing for first-level licensure exams. DPPs lasted a median of 7 weeks (IQR 6–8 weeks), during which students reported spending 70 h/week (IQR 56–80 h/week) studying. Students used a median of 8 study resources (IQR 7–10). Most (78%) reported being very or extremely confident in the resources they selected for exam preparation.

### Changes in student activities and wellness

**Fig. 1:**
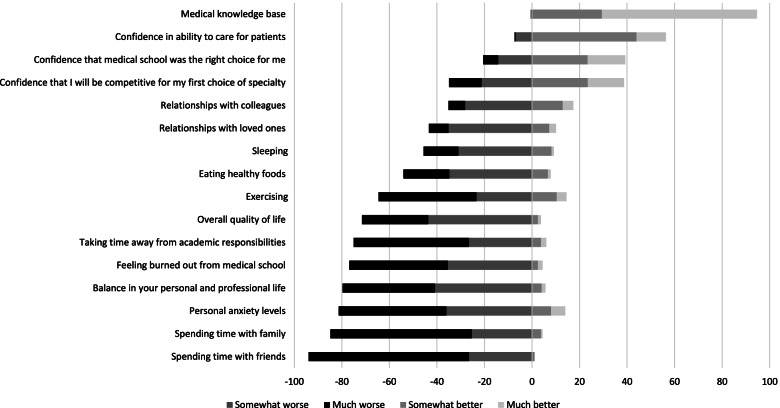
Percentages of 316 second-year students across 4 U.S. medical schools who reported changing for better or worse with their dedicated preparation period for first-level licensure exams. ^a^ Responses measured along a 5-point Likert-type scale. “No change” responses are not shown. Percentages refer to the absolute value of proportion of students experiencing the change

Figure 1 illustrates responses to the 16 items we used to measure changes attributed to students’ DPPs. Most students felt that 2 outcomes improved: medical knowledge base (95%) and confidence in ability to care for patients (56%). Most reported 9 outcomes worsened, including overall quality of life (72%), feeling burned out from medical school (77%), and personal anxiety levels (81%). Most also reported burnout (71%) and feelings of depression (52%) weekly or more often.

Rates for 5 outcomes showed statistically significant differences across the 4 schools (*p* < .0027): eating healthy foods, confidence that medical school was the right choice, confidence in being competitive for their specialty, relationships with colleagues, and relationships with loved ones. No statistically significant differences were seen in rates across schools for the other 13 items.

### Study time and wellness

Relationships among hours per week studying and the 18 outcomes items are shown in Table 1. More time studying per week was significantly associated with 3 items after adjusting for clustering in schools (*p* < .0027): better medical knowledge base, worse time with family, and worse time away from academic activities.

**Table 1:** Relationships among professional and wellness factors and quartiles of hours per week studying during dedicated preparation periods (DPPs)

	Quartile 1^**a**^	Quartile 2	Quartile 3	Quartile 4	***p***^**b**^
N	81	135	57	49	n/a
Hours per week studying during DPPs, median (IQR)	42 (20-50)	65 (60-70)	80 (75-80)	90 (84-100)	n/a
	**Percent responding got better**				
Medical knowledge base	91%	94%	98%	100%	<. 001
Confidence to care for patients	61%	52%	56%	62%	0.909
Confidence in medical school	48%	37%	39%	34%	0.477
Confidence to get first choice of specialty	39%	40%	28%	49%	0.462
	**Percent responding got worse**				
Sleeping	36%	47%	54%	49%	0.008
Eating healthy foods	59%	49%	53%	62%	0.716
Exercising	65%	54%	77%	77%	0.082
Time with friends	90%	94%	96%	98%	0.118
Time with family	81%	83%	86%	94%	<. 001
Time away from academic work	69%	74%	79%	83%	0.002
Relationships with colleagues	33%	37%	32%	38%	0.702
Relationships with loved ones	41%	40%	51%	45%	0.496
Balance in personal and professional life	76%	79%	86%	79%	0.445
Personal anxiety levels	74%	83%	89%	79%	0.564
Burned out from medical school	75%	76%	79%	79%	0.559
Overall quality of life	61%	73%	74%	81%	0.027
	**Percent responding weekly or more often**				
Burned out from work and studying	59%	77%	77%	68%	0.131
Feeling down, depressed, or hopeless	40%	58%	56%	51%	0.311

^a^Quartiles for hours per week were created using Stata xtile function and are not evenly distributed due to large numbers of students reporting the same values at the upper or lower bound of a quartile

^b^*p* values are unadjusted and correspond to logistic regressions adjusting for clustering within schools. Bonferroni correction for 18 comparisons requires *p* = .0027 to achieve significance at alpha = .05

*IQR* Interquartile Range, *DPP* Dedicated Preparation Period

### Spending and perceived financial strain and outcomes

Students reported spending a median of $650 (IQR $440–1000) on resources or programs to prepare for their exam. A total of 25% (*n* = 80) reported that paying for preparation materials strained their finances “a lot” or “extremely.” Greater amount spent for preparation was associated with greater perceived financial strain (*p* < .001).

Significant associations (*p* < .0027) were found with increased amount spent on preparation resources and two outcomes items: worse exercising and overall quality of life. Greater strain on finances was associated with less favorable responses to 11 of the 18 outcomes items (*p* < .0027) (Table 2).

**Table 2:** Relationships among professional and wellness factors and actual spending and perceived financial strain

	Quartiles of spending^**a**^					Perceived financial strain					
	1	2	3	4	*p*^b^	Not at all	A little	Somewhat	A lot	Extremely	*p*^b^
n	80	82	102	52	n/a	47	81	109	64	15	n/a
Out of pocket payment in $: Median (IQR)	250 (170-300)	500 (500-600)	1000 (800-1000)	1500 (1200-2000)	n/a	500 (250-800)	560 (300-800)	700 (440–1000)	1000 (600-1200)	1000 (700-1440)	n/a
**Percent responding got better**											
Medical knowledge base	95%	95%	94%	94%	0.622	100%	94%	93%	97%	87%	0.183
Confidence to care for patients	54%	61%	56%	54%	0.921	62%	57%	60%	52%	33%	0.192
Confidence in medical school	36%	37%	42%	42%	0.101	38%	51%	39%	31%	20%	0.079
Confidence to get first choice of specialty	36%	37%	41%	41%	0.692	51%	44%	39%	25%	20%	<.001
**Percent responding got worse**											
Sleeping	40%	48%	43%	56%	0.039	23%	36%	45%	66%	87%	<.001
Eating healthy foods	49%	48%	60%	62%	0.049	36%	47%	57%	64%	87%	0.006
Exercising	51%	66%	69%	75%	<.001	47%	59%	71%	72%	73%	<.001
Time with friends	94%	94%	91%	100%	0.370	94%	93%	93%	97%	100%	0.436
Time with family	80%	87%	82%	94%	0.011	81%	85%	79%	95%	93%	<.001
Time away from academic work	78%	73%	75%	75%	0.823	72%	72%	74%	80%	87%	0.135
Relationships with colleagues	31%	35%	36%	38%	0.588	26%	33%	36%	36%	67%	<.001
Relationships with loved ones	33%	46%	48%	46%	0.056	26%	35%	49%	52%	73%	0.003
Balance in personal and professional life	76%	82%	78%	85%	0.216	74%	72%	81%	88%	100%	<.001
Personal anxiety levels	83%	82%	77%	87%	0.891	68%	74%	87%	86%	100%	<.001
Burned out from medical school	76%	73%	75%	88%	0.090	72%	69%	79%	81%	100%	0.002
Overall quality of life	64%	73%	73%	79%	<.001	66%	52%	77%	88%	87%	<.001
**Percent responding weekly or more often**											
Burned out from work and studying	64%	74%	70%	81%	0.175	57%	63%	73%	84%	87%	<.001
Feeling down, depressed, or hopeless	48%	54%	50%	62%	0.016	32%	42%	55%	67%	87%	<.001

^a^Quartiles for amount spent were created using Stata xtile function and are not evenly distributed due to large numbers of students reporting the same values at the upper or lower bound of a quartile

^b^*p* values are unadjusted and correspond to logistic regressions adjusting for clustering within schools. Bonferroni correction for 18 comparisons requires *p* = .0027 to achieve significance at alpha = .05

*IQR* Interquartile Range

### Life events during DPPs

62/314 (20%) respondents reported experiencing a significant life event that impacted their ability to study during their DPPs, with something that happened to a family member or friend as the most common type of event (39/62 = 63%), followed by personal illness (15/62 = 24%), academic/extracurricular commitments (5/62 = 8%), and financial matters (4/62 = 6%). Examples of life events described by respondents included illnesses or death of loved ones; divorce or break-up; responsibility to work to provide for one’s family; new diagnosis, work-up, or treatment of one’s own illness; and pressure to complete abstracts or other academic projects during the DPP. Compared to those who did not report having a life event, those who reported having a life event had significantly worse changes to exercising (61% vs 79%, *p* < .001), balance in their personal and professional life (78% vs 87%, *p* < .001), and personal anxiety levels (69% vs 82%, *p* < .001) compared to those who did not report having a life event, but had no other statistically significant differences in the other 15 outcomes.

### Qualitative items on personal and professional influence of DPPs

Analysis of students’ descriptions of how preparation for their exams influenced them personally and professionally revealed the following themes: (1) opportunity for synthesis of medical knowledge, (2) exercising the endurance and self-discipline required for professional practice, (3) dissonance between exam preparation resource content, formal curriculum, and professional values, (4) isolation, deprivation, and anguish in a competition for the highest possible score, and (5) effects on well-being extending beyond DPPs. Quotes that illustrate each theme are included in Table 3.

**Table 3:** Themes and illustrative quotes from students’ descriptions of how exam preparation influenced them personally and professionally

Theme	Quote(s)
Opportunity for synthesis of medical knowledge	*“It was a great final review of all the material I had learned over the first two years of medical school and my knowledge base grew a lot.”* *“I appreciated being able to only focus on the exam and not having to deal with other random assignments or responsibilities, and I loved being able to finally synthesize and understand the content we had learned over the past two years. It reinforced my love for medical knowledge.”*
Exercise of endurance and self-discipline required for professional practice	*“My preparation made me realize that dedication it takes to practice medicine, having to be willing to test myself and learn new information every day in order to master the breadth of medical knowledge.”* *“The routine for studying every day was definitely an exercise of discipline and trusting the process …*. *the discipline and motivation that helped get me through board prep will hopefully stick with me in my career as a physician.”*
Dissonance between exam preparation resource content, formal curriculum, and professional values	*“Step prep tore down all the faith I have in medicine …. I don’t think that knowing all the biochemical pathways and all the minute details like that are necessarily going to make us better doctors.”* *“I feel that the importance of Step 1 scores is inflated in a way that benefits students who skip lectures and “social medicine” learning, and focus solely on memorizing First Aid. This isn’t the way that medical education should be. I don’t see how high performance on Step 1 could possibly be an adequate measure of how successful someone will be as a doctor, but it seems that this is the favored way to evaluate our value as students and future residents.”*
Isolation, deprivation, and anguish in a competition for the highest possible score	*“Step study time was a dark time where your whole life revolves around getting one score with the feeling that failing to achieve that score will prevent you from doing what you want. Your mental health suffers inevitably. Your relationships with family friends suffer. There is a great deal of anxiety as the test date approaches and you begin comparing your progress to colleagues.”* *“Studying for Step 1 was emotionally demanding, and was the most isolating experience of my life. For my goal score, I knew that I had to give up a lot that was important to me. This meant minimizing contact with my close friends, and only maintaining my relationship with my partner and parents. I discontinued exercise, and did not take many breaks, which I regret.”* *“Every time I would see my classmates in the library, I felt immense anxiety and nausea. Some of my classmates would be in the library 12-14 h a day and it was just extremely disheartening. I never got to spend time with friends and the time I didn’t spend studying was time spent feeling guilty for not studying. Every question I got wrong made me feel worthless.”*
Effects on well-being extending beyond DPPs	*“I did not realize until it was over and I began my clinical rotations how much it was affecting me personally. I was having insomnia in the months before that has completely disappeared, as well as mild symptoms of depression that are significantly better now.”* *“I have never been so burned out before during medical school. This feeling of being burned out has carried over to my rotations. I currently still feel burned out.”* *“I felt like I changed as a person after dedicated. Now I jump to conclusions faster about people and am generally more impatient. I also feel unhealthy, both physically and mentally. I feel like I am constantly bearing the weight of high expectations, my patients, and the fear of failure.”*

*DPP* Dedicated Preparation Period

Students’ descriptions of what it was like to have the exam behind them included feeling “relieved,” “numb,” “tired,” “terrible,” and “hopeful.” Students rested, caught up with friends and family, and traveled in the period immediately after their exams.

## Discussion

This is the first multi-institution study to examine medical student well-being during dedicated preparation periods (DPPs) for high-stakes exams. Our findings can be used to inform recommendations for students and schools to make exam preparation less stressful and to add relevant evidence to licensure exam reform discussions.

Students begin medical school with well-being ratings that are similar or higher than their peers’ [41], but subsequently medical students, residents, and physicians have worse well-being than their non-medical colleagues [42, 43]. Exactly when the decline in well-being starts is not clear. Available data from the U.S. have suggested that well-being may remain stable during the first year of medical school [44] and worsen during the second year [45, 46], resulting in approximately 35–50% of medical students having symptoms of burnout and 25% having symptoms of depression [46–48]. USMLE Step 1, and to a lesser degree, COMLEX Level 1, have been invoked as worsening medical student well-being although supporting evidence has been limited. At one school, nearly three-fourths of students surveyed at the start of their clerkships reported burnout [49], and at another school, students surveyed shortly after their DPP reported burnout (79%), and feelings of anxiety or depression (61%), which they attributed to preparation for USMLE Step 1 [12]. Our estimates of burnout and depressive symptoms across four schools were substantially higher than the rates for burnout and depression during medical school in general and more consistent with reports from students who recently completed their DPPs. We further found that most other measures of well-being were reported to worsen during DPPs. Our estimates provide a new level of detail that characterizes student well-being and the specific healthy activities that may become compromised during their DPPs. These data may be useful in future studies that make comparisons or attempt to monitor interventions aimed at maintaining wellness during DPPs. As more countries implement standardized national licensure exams [50], they may be more attuned to students’ experiences in preparing for them.

Students’ statements about how DPPs influence them may provide insight into what occurs to students’ professional identity formation during DPPs. High-stakes exams have been implicated in eroding trust in medical education institutions and undermining traditional professional values [51]. Our study suggests that these processes are exacerbated during DPPs, while competition and the pursuit of maximum knowledge at the expense of self-care are incentivized. This may tacitly reinforce a traditional stoic physician stereotype that a growing number of wellness programs [52] seek to counteract. While many students may manage DPPs as a time of temporary imbalance and recover completely from them, our findings indicate that some may have lasting changes. Should a decline in the well-being be precipitated or exacerbated during DPPs and continue through students’ training, this could influence relationships in their personal and professional lives, and perpetuate a professional culture that makes it difficult to address the systemic factors that contribute to the epidemic of provider distress.

Mitigating educational debt has been proposed as a way to improve medical student well-being [53]. Commentaries on Step 1 have also suggested that the cost of exam preparation worsens the learning environment and ultimately contributes to inequities and physician workforce maldistribution [4, 5, 54]. Consistent with previous estimates [4, 5] and empirical work [12, 55], we found that students incur significant out-of-pocket expenses for exam preparation resources, despite being given preparation resources that have been paid for by their institutions. However, the level of student spending on exam preparation was small compared to the approximate median U.S. medical student debt of $200,000, and less than the price of taking each licensing exam. Evidence linking student spending on medical education and their well-being is mixed [56–60]. Our finding that financial strain was more closely associated with worse well-being than was actual spending is consistent with a qualitative study indicating that students experience debt in a variety of ways and that perception of debt may be more important than debt amount [61]. More work is needed to understand the relationship between educational spending and perceived financial strain. In the meantime, monitoring students’ financial strain may be more beneficial than tracking their spending amount. Also, as purchasing exam preparation materials has become an educational expense that all students preparing for their exams bear, schools may consider the benefits of offering needs-based financial assistance for exam preparation, as they do for tuition, to enhance both well-being and equity.

Important limitations must be considered when reviewing our findings. First, our response rate varied across schools, and overall, respondents were less than a majority of those in our sample. While there was consistency across the four schools in many of our findings, our ability to generalize across all students in our sample and to medical schools in general is limited. Second, our measures were self-reported, and we had an inability to adjust for student wellness before they began their DPPs. While we attempted to overcome this by asking for changes attributed to DPPs, responses are subject to bias. Third, while surveys were sent shortly after students completed their DPPs to improve their recall accuracy, not all students took their exams at the same time. Some students knew their exam scores and some did not, which could bias their perceptions of how well-being was influenced during DPPs. Fourth, while we attempted to shed light on student experiences with DPPs by measuring multiple dimensions of wellness and capturing students’ qualitative comments, there were inevitably important aspects of wellness that we did not evaluate and which warrant further investigation. Likewise, there are other variables that may contribute to changes in well-being during DPPs’ that we did not measure (e.g. expectations for their own performance) and could be included in future study. Finally, whether changes in well-being during DPPs are specific only to the particular circumstances of preparing for USMLE Step 1 or COMLEX Level 1, or would generalize to DPPs used for other high-stakes exams is unknown. All indications suggest that scores from Step 2 CK and COMLEX Level 2 will be used by residency programs just as first-level licensure exam scores had been and students may engage in DPPs for those exams that resemble those for students in our study; our methods may inform studies of preparation for those and other high-stakes licensure exams.

## Conclusions

Every year, approximately half of the applicants to U.S. medical schools are turned away. The students who matriculate typically have demonstrated academic and personal excellence and an enthusiasm for learning medicine in service to patients and society. Taking time to develop and consolidate one’s medical knowledge is necessary to foster the expertise that all practicing physicians must possess and apply to their patient care. Our study has important implications for how students should develop and consolidate their knowledge foundation. Learning medicine can be gratifying and should be perceived as a privilege of participation in the profession. There is significant risk to student well-being and potentially to the profession if DPPs for high-stakes exams continue in their current forms.

Indeed, we should question whether there should be DPPs at all. Physicians must develop habits of continuous learning, and greater emphasis on continuous assessment that spaces learning over a longer period would be more effective at gaining knowledge than intense DPPs. Greater alignment of curriculum and licensure exam content may also help to make DPPs obsolete. In the meantime, strategies at a system-level to relieve the emphasis placed on a single exam score should be implemented to alleviate the stress students feel during DPPs. Schools should also recognize that many students placed into DPPs for required high-stakes exams experience them as a time of deprivation and suffering, and seek to offer preventative and supportive resources that mitigate DPP-related harms.

## Supplementary Information

**Additional file 1.**

## Data Availability

The datasets generated and analysed during the current study are not publicly available because they contain medical student information that they did not consent to have shared publicly at the individual level but aspects of the dataset may be available from the corresponding author on reasonable request.

## References

[1] Kappelman MM (1983). The impact of external examinations on medical education programs and students. J Med Educ.

[2] Wagoner NE, Gray GT (1979). Report on a survey of program directors regarding selection factors in graduate medical education. J Med Educ.

[3] NRMP. Results of the 2008 NRMP Program Director Survey [Internet]. Available from: https://mk0nrmp3oyqui6wqfm.kinstacdn.com/wp-content/uploads/2013/08/programresultsbyspecialty.pdf

[4] Chen DR, Priest KC, Batten JN, Fragoso LE, Reinfeld BI, Laitman BM (2019). Student perspectives on the “step 1 climate” in preclinical medical education. Acad Med.

[5] Carmody JB, Rajasekaran SK. On step 1 mania, USMLE score reporting, and financial conflict of interest at the National Board of medical examiners. Acad Med. 2020;1332–7. 10.1097/ACM.0000000000003126 31850948

[6] Chaudhry HJ, Katsufrakis PJ, Tallia AF (2020). The USMLE step 1 decision: an opportunity for medical education and training. JAMA.

[7] COMLEX-USA Level 1 to Eliminate Numeric Scores [Internet]. Available from: https://www.nbome.org/news/comlex-usa-level-1-to-eliminate-numeric-scores/

[8] Makhoul AT, Pontell ME, Kumar NG, Drolet BC (2020). Objective measures needed-program directors’ perspectives on a pass/fail USMLE step 1. N Engl J Med.

[9] Rashid H, Coppola KM, Lebeau R (2020). Three Decades Later: A Scoping Review of the Literature Related to the United States Medical Licensing Examination. Acad Med.

[10] Prober CG, Kolars JC, First LR, Melnick DE (2016). A plea to reassess the role of United States medical licensing examination step 1 scores in residency selection. Acad Med.

[11] Andolsek KM (2019). One small step for step 1. Acad Med.

[12] Cortes-Penfield NW, Khazanchi R, Talmon G. Educational and Personal Opportunity Costs of Medical Student Preparation for the United States Medical Licensing Examination Step 1 Exam: A Single-Center Study. Cureus. 2020;12(10):e10938. 10.7759/cureus.10938 PMC7660126 33194500

[13] McDonald FS, Jurich D, Duhigg LM, Paniagua M, Chick D, Wells M, et al. Correlations between the USMLE step examinations, American College of Physicians in-Training Examination, and ABIM internal medicine certification examination. Acad Med. 2020;95(9):1388–95. 10.1097/ACM.0000000000003382 32271224

[14] Richards BF, Cariaga-Lo L (1994). Curriculum type and sophomore students’ preparation time for the Usmle step 1 examination. Eval Health Prof.

[15] Kumar AD, Shah MK, Maley JH, Evron J, Gyftopoulos A, Miller C. Preparing to take the USMLE step 1: a survey on medical students ’ self-reported study habits. Postgrad Med J. 2015;91(1075):257–61. 10.1136/postgradmedj-2014-133081 25910497

[16] Giordano C, Hutchinson D, Peppler R (2016). A predictive model for USMLE step 1 scores. Cureus..

[17] Tanenbaum EJ, Johnson JH, Jordan E, Cottral J, Tenore C, Burton WB, et al. An Effective Evidence-Based Student Run Near-Peer Support Group for the USMLE Step 1 Exam. Med Sci Educ [Internet]. 2016:691–9. 10.1007/s40670-016-0334-8.

[18] Burk-Rafel J, Santen SA, Purkiss J (2017). Study behaviors and USMLE step 1 performance: implications of a student self-directed parallel curriculum. Acad Med.

[19] Baños JH, Pepin ME, Van Wagoner N (2017). Class-wide access to a commercial step 1 question Bank during preclinical organ-based modules. Acad Med [Internet].

[20] Schwartz LF, Lineberry M, Park YS, Kamin CS, Hyderi AA (2017). Development and evaluation of a student-initiated test preparation program for the USMLE step 1 examination. Teach Learn Med [internet].

[21] Parry S, Pachunka J, Beck Dallaghan GL (2019). Factors predictive of performance on USMLE step 1: do commercial study aids improve scores?. Med Sci Educ.

[22] Jackson F, Duane E, Harmon R, Kollar RA, Rainville NM, Smith RM. Resources that improve medical board licensing examination performance. Cureus. 2019;11(10):e5927. 10.7759/cureus.5927 PMC6857833 31788384

[23] Seal ZA, Koek W, Sharma R (2020). Correlation of medical college admission test scores and self-assessment materials with the United States medical licensing examination step 1 performance. Cureus..

[24] Thadani RA, Swanson DB, Galbraith RM. A preliminary analysis of different approaches to preparing for the USMLE step 1. Acad Med. 2000;75(10 Suppl):S40–2. 10.1097/00001888-200010001-00013 11031169

[25] Werner LS, Bull BS. The effect of three commercial coaching courses on step one USMLE performance. Med Educ. 2003;37(6):527–31. 10.1046/j.1365-2923.2003.01534.x 12787375

[26] Zhang C, Rauchwarger A, Toth C (2005). Student USMLE step 1 preparation and performance. Adv Health Sci Educ.

[27] Alcamo AM, Davids AR, Way DP, Lynn DJ, Vandre DD (2010). The impact of a peer-designed and -led USMLE step 1 review course: improvement in preparation and scores. Acad Med [Internet].

[28] Strowd RE, Beard HR, Gorney B, Russell GB, Lambros A (2013). The impact of process-oriented preparation on high-stakes testing in medical school. Med Sci Educ.

[29] Vora A, Maltezos N, Alfonzo L, Hernandez N, Calix E, Fernandez MI (2013). Predictors of scoring at least 600 on COMLEX-USA level 1: successful preparation strategies. J Am Osteopath Assoc.

[30] Bonasso P, Lucke-Wold B III, Reed Z, Bozek J, Cottrell S. Investigating the impact of preparation strategies on USMLE step 1 performance. MedEdPublish. 2015;4(1):5. 10.15694/mep.2015.004.0005 PMC4975378 27500163

[31] Deng F, Gluckstein JA, Larsen DP (2015). Student-directed retrieval practice is a predictor of medical licensing examination performance. Perspect Med Educ.

[32] Roscoe LJ (2009). Wellness: a review of theory and measurement for counselors. J Couns Dev.

[33] Liu R, Carrese J, Colbert-Getz J, Geller G, Shochet R (2015). “Am I cut out for this?” understanding the experience of doubt among first-year medical students. Med Teach [Internet].

[34] Miller RE, Kelleher M, Duckett A, O’Rourke P, Yen M-S, Call SA (2020). Time allocation and well-being in internal medicine residents: a multi-institutional cross-sectional survey. Am J Med.

[35] West CP, Shanafelt TD, Kolars JC (2011). Quality of life, burnout, educational debt, and medical knowledge among internal medicine residents. JAMA.

[36] West CP, Dyrbye LN, Satele DV, Sloan JA, Shanafelt TD (2012). Concurrent validity of single-item measures of emotional exhaustion and depersonalization in burnout assessment. J Gen Intern Med.

[37] Cook AF, Arora VM, Rasinski KA, Curlin FA, Yoon JD (2014). The prevalence of medical student mistreatment and its association with burnout. Acad Med [Internet].

[38] Dyrbye LN, Schwartz A, Downing SM, Szydlo DW, Sloan JA, Shanafelt TD (2011). Efficacy of a brief screening tool to identify medical students in distress. Acad Med.

[39] Greenburg DL, Durning SJ, Cruess DL, Cohen DM, Jackson JL (2010). The prevalence, causes, and consequences of experiencing a life crisis during medical school. Teach Learn Med.

[40] LaDonna KA, Taylor T, Lingard L (2018). Why open-ended survey questions are unlikely to support rigorous qualitative insights. Acad Med.

[41] Brazeau CMLR, Shanafelt T, Durning SJ, Massie FS, Eacker A, Moutier C (2014). Distress among matriculating medical students relative to the general population. Acad Med [Internet].

[42] Dyrbye LN, West CP, Satele D, Boone S, Tan L, Sloan J (2014). Burnout among U.S. medical students, residents, and early career physicians relative to the general U.S. Population Acad Med [Internet].

[43] Shanafelt TD, West CP, Sinsky C, Trockel M, Tutty M, Satele DV (2019). Changes in burnout and satisfaction with work-life integration in physicians and the general US working population between 2011 and 2017. Mayo Clin proc [internet].

[44] Jordan RK, Shah SS, Desai H, Tripi J, Mitchell A, Worth RG (2020). Variation of stress levels, burnout, and resilience throughout the academic year in first-year medical students. PLoS One [Internet].

[45] Santen SA, Holt DB, Kemp JD (2010). Burnout in medical Students : examining the prevalence and associated factors. South Med J.

[46] Rotenstein LS, Ramos MA, Torre M, Segal JB, Peluso MJ, Guille C (2016). Prevalence of depression, depressive symptoms, and suicidal ideation among medical students: a systematic review and meta-analysis. JAMA.

[47] Dyrbye L, Shanafelt T (2016). A narrative review on burnout experienced by medical students and residents. Med Educ.

[48] Taking Action Against Clinician Burnout. Taking action against clinician burnout: National Academies Press; 2019. 31940160

[49] Mazurkiewicz R, Korenstein D, Fallar R, Ripp J (2012). The prevalence and correlations of medical student burnout in the pre-clinical years: a cross-sectional study. Psychol Health Med.

[50] Price T, Lynn N, Coombes L, Roberts M, Gale T, de Bere SR (2018). The international landscape of medical licensing examinations: a typology derived from a systematic review. Int J Heal Policy Manag [Internet].

[51] Hafferty FW, O’Brien BC, Tilburt JC (2020). Beyond high-stakes testing: learner trust, educational commodification, and the loss of medical school professionalism. Acad Med.

[52] Dyrbye LN, Sciolla AF, Dekhtyar M, Rajasekaran S, Allgood JA, Rea M (2019). Medical school strategies to address student well-being: a National Survey. Acad Med.

[53] Dyrbye LN, Lipscomb W, Thibault G (2020). Redesigning the learning environment to promote learner well-being and professional development. Acad Med.

[54] Cangialosi PT, Chung BC, Thielhelm TP, Camarda ND, Eiger DS. Medical Students’ Reflections on the Recent Changes to the USMLE Step Exams. Acad Med. 2021;96(3):343–8. 10.1097/ACM.0000000000003847 PMC8081295 33208676

[55] Bhatnagar V, Diaz SR, Bucur PA (2019). The cost of board examination and preparation: an overlooked factor in medical student debt. Cureus..

[56] Dyrbye LN, Thomas MR, Massie FS, Power DV, Eacker A, Harper W (2008). Burnout and suicidal ideation among U.S. medical students. Ann Intern Med [Internet].

[57] Dyrbye L, Sloan J, Shanafelt TD (2009). Is there a connection between high educational debt and suicidal ideation among medical students?. Ann Intern Med [Internet].

[58] Rohlfing J, Navarro R, Maniya OZ, Hughes BD, Rogalsky DK. Medical student debt and major life choices other than specialty. Med Educ Online. 2014;19:1–10. 10.3402/meo.v19.25603 PMC4229497 25391976

[59] Jackson ER, Shanafelt TD, Hasan O, Satele DV, Dyrbye LN (2016). Burnout and alcohol abuse/dependence among U.S. medical students. Acad Med.

[60] Dyrbye LN, Wittlin NM, Hardeman RR, Yeazel M, Herrin J, Dovidio JF (2019). A prognostic index to identify the risk of developing depression symptoms among U.S. medical students derived from a national, four-year longitudinal study. Acad Med.

[61] Phillips JP, Wilbanks DM, Salinas DF, Doberneck DM (2016). Educational debt in the context of career planning: a qualitative exploration of medical student perceptions. Teach Learn Med [Internet].

