# Supplementary material for: Student well-being during dedicated preparation for USMLE Step 1 and COMLEX Level 1 exams
Source: BMC Med Educ. 2022 Jan 4;22:16. doi: 10.1186/s12909-021-03055-2 (PMC8728922; doi:10.1186/s12909-021-03055-2)
Supplement: Supplementary file 1 — Additional file 1. [file 12909_2021_3055_MOESM1_ESM.docx]

Survey instrument

Start of Block: Default Question Block

Q39 Thank you for your interest in this study. Your completion of this survey will serve as your consent to be in this research study. Your personal data will be kept confidential.

Thanks Again!

Sean Tackett, MD, MPH

Johns Hopkins Bayview Medical Center

Q92 So far during medical school, how important has each of the following types of resources been to building your medical knowledge foundation?

|  | Unimportant (5) | Slightly important, used a little bit (4) | Fairly important, used intermittently (3) | Very important, used often (2) | Essential, used frequently (1) |
| --- | --- | --- | --- | --- | --- |
| Commercial resources (e.g. Pathoma, Anki, etc) (1) |  |  |  |  |  |
| Institution-produced resources (e.g. faculty lecture slides and syllabi) (2) |  |  |  |  |  |
| Student-produced resources (e.g. peer note-taking) (3) |  |  |  |  |  |
| Other (please specify) (4) |  |  |  |  |  |

Q93 Before your dedicated preparation for Step 1, how often did you attended your school’s:

|  | Almost never (1) | Occasionally (2) | Somewhat often (3) | Often (4) | Most of the time (5) |
| --- | --- | --- | --- | --- | --- |
| In-person pre-clerkship courses/lectures (1) |  |  |  |  |  |
| Virtual pre-clerkship courses/lectures (e.g., podcast or video) (2) |  |  |  |  |  |

| 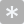 |
| --- |

Q94 Before your dedicated preparation for USMLE Step 1, about how many hours per week did you devote to studying for USMLE Step 1?

________________________________________________________________

| Page Break |  |
| --- | --- |

| 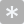 |
| --- |

Q8 Approximately how many weeks did you focus on dedicated preparation for the USMLE Step 1 exam?

________________________________________________________________

| 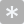 |
| --- |

Q9 During dedicated preparation for USMLE Step 1, about how many hours per week did you devote to studying?

________________________________________________________________

| 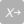 |
| --- |

Q11 How important was each of the following resources during your preparation for USMLE Step 1?

|  | N/A: I've never heard of this resource (6) | Unimportant, I heard of this but didn't try it (5) | Slightly important, used a little bit (4) | Fairly important, used intermittently (3) | Very important, used often (2) | Essential, used frequently (1) |
| --- | --- | --- | --- | --- | --- | --- |
| Amboss (30) |  |  |  |  |  |  |
| Anki (6) |  |  |  |  |  |  |
| Becker (28) |  |  |  |  |  |  |
| Boards and Beyond (22) |  |  |  |  |  |  |
| Cramfighter (11) |  |  |  |  |  |  |
| Doctors in Training (14) |  |  |  |  |  |  |
| Firecracker (5) |  |  |  |  |  |  |
| First Aid for the USMLE Step 1 (4) |  |  |  |  |  |  |
| Goljan Audio (25) |  |  |  |  |  |  |
| Kaplan Qbank (10) |  |  |  |  |  |  |
| Lecturio (23) |  |  |  |  |  |  |
| Memorang (26) |  |  |  |  |  |  |
| Osmosis (1) |  |  |  |  |  |  |
| Pathoma (3) |  |  |  |  |  |  |
| Picmonic (7) |  |  |  |  |  |  |
| Quizlet (27) |  |  |  |  |  |  |
| Sketchy (8) |  |  |  |  |  |  |
| USMLE-Rx question bank (9) |  |  |  |  |  |  |
| USMLE World (2) |  |  |  |  |  |  |
| Commercial tutoring programs (e.g. Med School Tutors, PASS) (29) |  |  |  |  |  |  |
| Syllabi or study materials provided by your school (19) |  |  |  |  |  |  |
| Study materials provided by other students (20) |  |  |  |  |  |  |
| Other (please specify) (21) |  |  |  |  |  |  |

Display This Question:

If How important was each of the following resources during your preparation for USMLE Step 1? [ First Aid for the USMLE Step 1 ] (Recode) < 5

Q44 Approximately how many "complete passes" did you make through First Aid before taking Step 1?

- < 0.5 complete passes (1)
- 0.5 complete passes (2)
- 1.0 complete passes (3)
- 1.5 complete passes (4)
- 2.0 complete passes (5)
- 2.5 complete passes (6)
- 3.0 complete passes (7)
- > 3.0 complete passes (8)

Display This Question:

If How important was each of the following resources during your preparation for USMLE Step 1? [ Amboss ] (Recode) < 5

| 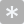 |
| --- |

Q40 Approximately how many questions  (new and repeated) did you complete from the Amboss question bank?

________________________________________________________________

Display This Question:

If How important was each of the following resources during your preparation for USMLE Step 1? [ USMLE World ] (Recode) < 5

| 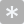 |
| --- |

Q117 Approximately how many questions  (new and repeated) did you complete from the USMLE World question bank? (U World has about 2400 questions in total)

________________________________________________________________

Display This Question:

If How important was each of the following resources during your preparation for USMLE Step 1? [ USMLE-Rx question bank ] (Recode) < 5

| 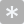 |
| --- |

Q115 Approximately how many questions  (new and repeated) did you complete from the USMLE-Rx question bank? (USMLE-Rx Step 1 Qmax has about 2200 questions in total)

________________________________________________________________

Display This Question:

If How important was each of the following resources during your preparation for USMLE Step 1? [ Osmosis ] (Recode) < 5

| 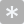 |
| --- |

Q42 Approximately how many questions (new and repeated) did you complete from the Osmosis question bank? (Osmosis has about 3300 questions for Step 1 in total)

________________________________________________________________

Display This Question:

If How important was each of the following resources during your preparation for USMLE Step 1? [ Kaplan Qbank ] (Recode) < 5

| 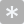 |
| --- |

Q43 Approximately how many questions (new and repeated) did you complete from the Kaplan Qbank? (Kaplan Qbank has about 2100 questions in total)

________________________________________________________________

| Page Break |  |
| --- | --- |

Q12 Did you take a NBME CBSSA or USMLE World practice test?

- Yes (1)
- No (2)

| Page Break |  |
| --- | --- |

Q97 How important was each of the following factors to you when choosing resources for USMLE Step 1 preparation?

|  | Not at all (1) | A little (2) | Somewhat (3) | Very (4) | Extremely (5) |
| --- | --- | --- | --- | --- | --- |
| Recommended by faculty or administrators at my school (1) |  |  |  |  |  |
| Recommended by students at my school (2) |  |  |  |  |  |
| Recommended by online sites for medical students (e.g. SDN, Reddit) (3) |  |  |  |  |  |
| Statements/advertisements that came from the product vendor (4) |  |  |  |  |  |
| Usefulness during a free trial (5) |  |  |  |  |  |
| School provided the resource for free (6) |  |  |  |  |  |
| Cost of resource (i.e. when paying out of pocket) (7) |  |  |  |  |  |
| Studies/evidence that showed the resource led to better exam scores (8) |  |  |  |  |  |

Q98 How confident were you in the resources that you ultimately selected to use for Step 1 preparation?

- Not at all (1)
- A little (2)
- Somewhat (3)
- Very (4)
- Extremely (5)

| Page Break |  |
| --- | --- |

| 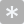 |
| --- |

Q62 What was the total amount ($) that you spent on resources/programs to prepare for USMLE Step 1?

________________________________________________________________

Q95 To what extent did paying for Step 1 preparation materials strain your finances?

- Not at all (1)
- A little (2)
- Somewhat (3)
- A lot (4)
- Extremely (5)

Q96 Are there any comments you'd like to share regarding the cost of Step 1 preparation materials (borne by you or your school)?

________________________________________________________________

________________________________________________________________

________________________________________________________________

________________________________________________________________

________________________________________________________________

| Page Break |  |
| --- | --- |

Q99 Did your school provide resources for you that were not offered to all students (e.g. those offered by student academic support or learning specialists)?

- Yes (23)
- No (24)

Display This Question:

If Did your school provide resources for you that were not offered to all students (e.g. those offer... = Yes

Q100 Which of the following supplementary resources did your school provide (please check all that apply)?

- Meetings with learning specialists (1)
- Individualized coaching by faculty (2)
- Peer-tutoring programs (3)
- Commercial resources (4)
- Other (please specify) (5) ________________________________________________

| Page Break |  |
| --- | --- |

Q101 To what extent did your preparation for USMLE Step 1 influence each of the following?

|  | Got much worse (1) | Somewhat worse (2) | No change (3) | Somewhat better (4) | Got much better (5) |
| --- | --- | --- | --- | --- | --- |
| Medical knowledge base (1) |  |  |  |  |  |
| Confidence in ability to care for patients (2) |  |  |  |  |  |
| Confidence that medical school was the right choice for me (3) |  |  |  |  |  |
| Confidence that I will be competitive for my first choice of specialty (4) |  |  |  |  |  |
| Relationships with colleagues (5) |  |  |  |  |  |
| Relationships with loved ones (6) |  |  |  |  |  |
| Balance in your personal and professional life (7) |  |  |  |  |  |
| Personal anxiety levels (8) |  |  |  |  |  |
| Feeling burned out from medical school (9) |  |  |  |  |  |
| Overall quality of life (10) |  |  |  |  |  |

Q102 Compared to what you usually do, how much did you engage in the following activities during dedicated preparation for USMLE Step 1?

|  | Much less (1) | Somewhat less (2) | About the same as usual (3) | Somewhat more (4) | Much more (5) |
| --- | --- | --- | --- | --- | --- |
| Sleeping (1) |  |  |  |  |  |
| Eating healthy foods (2) |  |  |  |  |  |
| Exercising (3) |  |  |  |  |  |
| Spending time with friends (4) |  |  |  |  |  |
| Spending time with family (5) |  |  |  |  |  |
| Taking time away from academic responsibilities (6) |  |  |  |  |  |

Q103 During your dedicated preparation for Step 1, please indicate how often you felt:

|  | Never (1) | Once a month or less (6) | A few times a month (2) | Once a week (3) | A few times a week (4) | Every day (5) |
| --- | --- | --- | --- | --- | --- | --- |
| Burned out from your work and studying (1) |  |  |  |  |  |  |
| Bothered by feeling down, depressed, or hopeless (2) |  |  |  |  |  |  |

Q104 During dedicated preparation for Step 1, did you experience a significant life event that you believe impacted your ability to study?

- Yes (23)
- No (24)

Display This Question:

If During dedicated preparation for Step 1, did you experience a significant life event that you bel... = Yes

Q105 Which of the following categories apply to the type of life event that you experienced (please check all that apply)?

- Something that happened to a family or friend (1)
- Financial matter (2)
- Personal illness (3)
- Academic/extracurricular commitments (e.g. research project, committee work, second degree program) (4)
- Other (5) ________________________________________________

Display This Question:

If During dedicated preparation for Step 1, did you experience a significant life event that you bel... = Yes

Q106 Could you please briefly describe what happened and how it impacted your preparation and performance for Step 1?

________________________________________________________________

________________________________________________________________

________________________________________________________________

________________________________________________________________

________________________________________________________________

| Page Break |  |
| --- | --- |

Q107 Would you please describe your personal strategy for preparing for USMLE Step 1?

________________________________________________________________

________________________________________________________________

________________________________________________________________

________________________________________________________________

________________________________________________________________

Q108 Would you please share how preparing for USMLE Step 1 influenced you personally and professionally?

________________________________________________________________

________________________________________________________________

________________________________________________________________

________________________________________________________________

________________________________________________________________

Q109 If you had to prepare for Step 1 all over again, what would you do differently?

________________________________________________________________

________________________________________________________________

________________________________________________________________

________________________________________________________________

________________________________________________________________

Q110 How did you feel after you completed your exam? What was it like to have the exam behind you?

________________________________________________________________

________________________________________________________________

________________________________________________________________

________________________________________________________________

________________________________________________________________

Q111 What did you do after you took your exam?

________________________________________________________________

________________________________________________________________

________________________________________________________________

________________________________________________________________

________________________________________________________________

Q112 Are there any other comments that you’d like to share?

________________________________________________________________

________________________________________________________________

________________________________________________________________

________________________________________________________________

________________________________________________________________

Q113 Do you already know your exam score?

- Yes (23)
- No (24)

Q47 May we contact you after this survey to ask additional questions?

- Yes (23)
- No (24)

End of Block: Default Question Block
